# Risk factors for testicular cancer: a case-control study in twins

**DOI:** 10.1038/sj.bjc.6690470

**Published:** 1999-06

**Authors:** A J Swerdlow, B L De Stavola, M A Swanwick, P Mangtani, N E S Maconochie

**Affiliations:** Department of Epidemiology and Population Health, London School of Hygiene and Tropical Medicine, Keppel Street, London WC1E 7HT, UK

**Keywords:** testicular cancer, twins, case-control

## Abstract

Early life and anthropometric risk factors for testicular cancer were examined in a case-control study in England and Wales in which affected male twins were compared with their unaffected male co-twins. Questionnaire data was obtained for 60 twin pairs. Significantly raised risk of testicular cancer occurred in twins who had longer arms and legs than their co-twin. There was a significant excess of testicular cancer reported in non-twin brothers, as well as in twin brothers, of cases. Risk was also significantly raised in relation to cryptorchidism. The results on limb length suggest that factors, perhaps nutritional, affecting growth before puberty, may be causes of testicular cancer. The results on risk in brothers add to evidence of a large genetic component in aetiology of the tumour. The risk associated with cryptorchidism in the twins accords with the hypothesis that cryptorchidism is causally associated with testicular cancer because it is a cause of the malignancy, rather than because the same maternal factors experienced in utero cause both conditions. © 1999 Cancer Research Campaign


*British Journal of Cancer* (1999) **80**(7), 1098–1102
© 1999 Cancer Research Campaign
Article no. bjoc.1998.0470

### Risk factors for testicular cancer: a case-control study in twins

**AJ Swerdlow, BL De Stavola, MA Swanwick, P Mangtani and NES Maconochie**

Department of Epidemiology and Population Health, London School of Hygiene and Tropical Medicine, Keppel Street, London WC1E 7HT, UK

**Summary** Early life and anthropometric risk factors for testicular cancer were examined in a case-control study in England and Wales in which affected male twins were compared with their unaffected male co-twins. Questionnaire data was obtained for 60 twin pairs. Significantly raised risk of testicular cancer occurred in twins who had longer arms and legs than their co-twin. There was a significant excess of testicular cancer reported in non-twin brothers, as well as in twin brothers, of cases. Risk was also significantly raised in relation to cryptorchidism. The results on limb length suggest that factors, perhaps nutritional, affecting growth before puberty, may be causes of testicular cancer. The results on risk in brothers add to evidence of a large genetic component in aetiology of the tumour. The risk associated with cryptorchidism in the twins accords with the hypothesis that cryptorchidism is causally associated with testicular cancer because it is a cause of the malignancy, rather than because the same maternal factors experienced in utero cause both conditions.

**Keywords:** testicular cancer; twins; case-control

Testicular cancer is the most common malignancy in young men in many Western countries, and is increasing in incidence. Despite epidemiological investigations over several decades its aetiology remains largely unknown. The peak of incidence of the tumour at around age 30 years and other features of its epidemiology suggest that aetiological factors operate early in life, either prenatally or in childhood and adolescence (Swerdlow, 1997). Investigation of exposures at such young ages is hampered, however, by the difficulty of gaining reliable information about them many years later by questions to adult patients. Case-control studies of malignancy in twins, comparing risk factors in affected twins with those in unaffected co-twins, give an unusual opportunity to investigate aetiological factors in early life, because *comparative* measures can be used to investigate factors that are usually difficult to recall exactly in order to enable comparison between cases and unrelated controls. We therefore conducted a case-control study of testicular cancer in twins in England and Wales.

### MATERIALS AND METHODS

The methods of identification of the subjects and collection of data from them have been described in detail elsewhere (Swerdlow et al, 1996, 1997). In brief, twin-born men with testicular cancer incident in England and Wales from 1971 to 1989 were identified by clerical linkage between national cancer registration files, birth registers and the National Health Service Central Register. All same-sex twin pairs identified in this way were contacted to administer questionnaires on zygosity (Torgersen, 1979; Mack et al, 1995) and potential risk factors for testicular cancer. These factors included perinatal variables, anthropometric measures at various ages, childhood diet and exercise, puberty, acne, childhood infections, other illnesses and malformations, and diseases and malformations in relatives. The risk factor information analysed was that reported in the questionnaires; no previously recorded information on these variables was available to us. When responses were contradictory or unclear, we attempted to resolve this by recontact with the subjects, usually by telephone.

For each risk factor, we examined the responses of the twin pair to questions about each other, in pairs in which both twins had replied, in order to determine whether responses from a single respondent about the two members of the pair would be reliable. When this proved to be so, we analysed data from the case and co-twin when both had replied, as well as from a single twin (about both of the pair) when he had been the sole respondent. When responses from subjects about their co-twins proved to be unreliable, we restricted the analyses to data from subjects about themselves. In analyses of comparative variables, pairs who gave contradictory responses to a question (e.g. each reported that they were the taller at a particular age) were excluded from analysis. In no instance did they constitute more than 5% of pairs. When one twin reported a difference between the pair for a comparative variable and the other twin reported none, the response stating a difference was used for the analysis. This was done because ‘the same’ height, for example, is likely to include small differences, whereas it is less likely that there would actually be no measurable difference. The results were similar, however, and the significant results remained so, if such pairs were omitted from the analysis.

Risks of testicular cancer in relation to various factors were assessed by matched case-control analysis, using conditional logistic regression (Breslow and Day, 1980). The twins with testicular cancer were the cases in these analyses and the co-twins

*Received 24 August 1998*
*Revised 14 November 1998*
*Accepted 18 November 1998*

*Correspondence to:* AJ Swerdlow

**Table 1** Relative risks in relation to birth order and anthropometric measures

| Risk factor | Case positive No. | Co-twin positive No. | Same No. | Not known or disagreed[a] No. | Odds ratio for factor positive (95% CI) |
|---|---|---|---|---|---|
| Born first | 31 | 29 | 0 | 0 | 1.1 (0.6–1.8) |
| Longer at birth | 12 | 13 | 12 | 23 | 0.9 (0.4–2.0) |
| Heavier at birth | 18 | 28 | 4 | 10 | 0.6 (0.4–1.2) |
| Taller at age 7 | 21 | 17 | 18 | 4 | 1.2 (0.6–2.3) |
| Heavier at age 7 | 22 | 24 | 13 | 1 | 0.9 (0.5–1.6) |
| Taller at age 11 | 23 | 18 | 17 | 2 | 1.3 (0.7–2.4) |
| Heavier at age 11 | 21 | 21 | 15 | 3 | 1.0 (0.5–1.8) |
| Taller at age 18 | 25 | 18 | 15 | 2 | 1.4 (0.8–2.5) |
| Heavier at age 18 | 20 | 27 | 9 | 4 | 0.7 (0.4–1.3) |
| Larger waist at age 18 | 20 | 23 | 11 | 6 | 0.9 (0.5–1.6) |
| Longer arms at age 18 | 19 | 7 | 26 | 8 | 2.7 (1.1–6.5)[b] |
| Longer legs at age 18 | 22 | 9 | 21 | 8 | 2.4 (1.1–5.3)[b] |

[a]In no instances were there more than three pairs who disagreed. [b]$P = 0.02$.

without testicular cancer were the controls. For pairs concordant for testicular cancer, the subject with malignancy incident earlier was taken as the case.

### RESULTS

We identified 194 cases of testicular cancer incident in twins during 1970–1989. Of these, 119 cases from 116 pairs, including three concordant pairs, were in male/male pairs and therefore eligible for the study. In two of these pairs both twins had died, and in three others the co-twin had died at a young age and no contact was attempted. Of the remaining 111 pairs, we obtained questionnaire replies from both twins in 37 instances and from one twin in 23 instances (16 cases and seven co-twins), leading to information on a total of 60 pairs. Based on the questionnaire data, 20 of these pairs were monozygotic (MZ) and 40 were dizygotic (DZ). The results described below are for MZ and DZ twins combined, except where otherwise stated. All but three of the pairs had lived together to at least age 10 years and all but seven to at least age 15 years.

Table 1 shows odds ratios (ORs) for various perinatal and anthropometric variables. Risk of testicular cancer was non-significantly raised for men who were taller than their co-twin, at each age investigated (7, 11 and 18 years), and significantly raised for men with longer arms and legs than their co-twin at age 18 (we did not enquire about limb length at younger ages). These associations derived from differences for DZ rather than MZ twins, although the zygosity-specific analyses were based on small numbers. The ORs for height, leg length and arm length at age 18 in DZ twins were 1.8 (95% confidence interval (CI) 0.9–3.8), 2.2 (1.0–5.2) and 3.4 (1.2–9.2) respectively.

We analysed the size of difference in height between the twins (not shown in Table 1), taking the mean of the reports of the two pair members when both had replied. Cases were, on average, taller than co-twins at each of ages 7, 11 and 18 years, by 0.50, 0.41 and 0.75 inches respectively. Similarly, cases had on average longer legs (by 0.40 inches) and arms (by 0.63 inches) at age 18 than control twins. It should be noted, however, that whereas the height values were based on responses for almost all pairs, those for leg and arm length were based on only 44 and 31 pairs respectively.

Questions about childhood diet (Table 2) showed no significant differences between cases and controls, although there was a near-significant raised risk, based on few responses of difference, for men who were reported to have eaten fewer vegetables than their co-twin. Questions about exercise in childhood, age at puberty (first shaving, voice breaking), acne and mumps (not in Table 2) showed no indication of an association with risk; for most of these factors, the great majority of subjects reported no difference between the case and co-twin.

Seven cases (five MZ, two DZ) and two controls (one MZ, one DZ) stated that they had had an undescended testis. One MZ pair

**Table 2** Relative risks in relation to childhood diet

| Dietary factor | Case consumed more No. | Co-twin consumed more No. | Same No. | Not known or disagreed[a] No. | Odds ratio for having consumed more (95% CI) |
|---|---|---|---|---|---|
| Fruit | 6 | 6 | 45 | 3 | 1.0 (0.3–3.1) |
| Fruit juice | 3 | 3 | 51 | 3 | 1.0 (0.2–5.0) |
| Vegetables | 4 | 12 | 40 | 4 | 0.3 (0.1–1.0) |
| Dairy products | 10 | 9 | 37 | 3 | 1.1 (0.4–2.7) |
| Eggs | 8 | 10 | 39 | 3 | 0.8 (0.3–2.0) |
| Red meat | 4 | 6 | 48 | 2 | 0.7 (0.2–2.4) |

[a]In no instance was there more than one pair who disagreed.

© 1999 Cancer Research Campaign was concordant for cryptorchidism, plus there were seven discordant pairs. There were no further instances of cryptorchidism reported solely second-hand, by the opposite twin. In pairs where both twins replied, reporting of cryptorchidism in the opposite twin, when that twin had already reported the condition in himself, was poor (under 50%). We therefore analysed the relative risk of testicular cancer in relation to cryptorchidism utilizing only reports by cases and controls about themselves in pairs where both had replied; this gave five discordant pairs, all with cryptorchidism in the case (i.e. OR = ∞), a significant ($P = 0.025$) excess.

We examined the birthweight for the seven pairs who were stated to be discordant for cryptorchidism. In one, the twins disagreed on who was heavier at birth, in another they were stated to be the same weight at birth, and in four of the remaining five pairs, the non-cryptorchid twin was the heavier at birth.

Six cases and three controls, including two pairs concordant for this, reported that they had had a groin hernia, in five cases and two controls without cryptorchidism. (Our questionnaire did not specifically ask whether these herniae were inguinal, since subjects might not understand this.) Cross-reporting of hernia by the opposite twin was poor, and again we therefore analysed only data from self-reports in pairs in which both twins replied. This gave six discordant pairs, five with hernia in the case, a non-significant relative risk of 5.0 ($P = 0.10$).

Three twin pairs (two MZ, one DZ) were concordant for testicular cancer. In addition, there were two DZ pairs who had a non-twin brother with the disease, and one (DZ) who had a cousin with it. We have analysed testicular cancer risks in the co-twins elsewhere (Swerdlow et al, 1997), although that analysis did not include as concordant one of the two MZ pairs described here because they did not meet the calendar period criteria for concordance in that study. For non-twin brothers, we estimate from the present data that the approximate relative risk of testicular cancer compared with rates expected from the general population (Office of Population Censuses and Surveys, 1985) was 26.2 (5.4–76.7; $P < 0.001$), on the conservative assumption that all non-twin brothers survived and were under follow-up to the time of interview.

There was a high frequency of cryptorchidism and hernia in the twin pairs who were concordant for testicular cancer: one of the concordant MZ pairs was also concordant for undescended testis and (groin) hernia; in the other MZ concordant pair, one of the twins had a hernia; and in the concordant DZ pair, one twin had an undescended testis. There were no other genitourinary malformations reported in the families of these concordant pairs, or in the two families in which the proband and a non-twin brother had testicular cancer. There were, however, several clusters of genitourinary malformations reported in families of other twin pairs in the study, discordant for testicular cancer. In one pair the case had an undescended testis (as well as testicular cancer) while the DZ co-twin had a son with an undescended testis and another son with bilateral (groin) herniae. In another pair, the case had a hernia, his father had a hernia, and he had three brothers (not his twin) with undescended testis or hernia. For another case, both his DZ co-twin and his father had a hernia, and for a further case his father had bilateral herniae plus a hydrocoele and his mother had a hernia.

### DISCUSSION

Twins have certain unique advantages in investigating aetiological factors in early life, but the disadvantage that it is difficult to ascertain sufficient numbers of cases for study, especially for an uncommon malignancy such as that of the testis. The present paper is, to our knowledge, the first to assess non-genetic risk factors for testicular cancer in twins, but the relatively small numbers of cases and controls that could be ascertained limit the conclusions.

An advantage of twin studies is that validation of responses (by use of the case's and co-twin's view on the same variable) is available for recall of variables such as height in childhood and age at puberty, for which none is available in a conventional case-control study with unrelated cases and controls. Non-response by eligible twin pairs is unlikely to have led to bias, because it is improbable that it was associated with twin/co-twin differences for the study variables (e.g. that non-response would be more (or less) likely in pairs where the case was the taller than in pairs where the co-twin was the taller).

The indication of a raised risk of testicular cancer in taller twins accords with evidence in this direction, but not strong, from four case-control studies of unrelated subjects (Brown et al, 1987; Swerdlow et al, 1989; UK Testicular Cancer Study Group, 1994; Gallagher et al, 1995), although not a fifth (Davies et al, 1990). A cohort study with fewer cases reported no association but did not publish further details (Whittemore et al, 1984). The twin approach has the advantage over studies of unrelated individuals that the twin pair, because they grew up together, are matched for many early life socio-economic related exposures that may be confounded with height. The risks of testicular cancer in relation to arm and leg length have not been examined previously, to our knowledge. Leg length, more than sitting height, has been found associated with risk of cancer overall and of some specific cancers in a study that did not examine risks of testicular cancer (Albanes et al, 1988).

Average adult height of men in Britain and other Western countries has been increasing for over a century (Tanner, 1989; Floud et al, 1990), and testicular cancer incidence likewise has been increasing for many decades, as long as data have been available (Swerdlow, 1997). Increased height mainly reflects prepubertal growth, probably consequent on greater protein intake (Proos, 1993), and in several countries has been almost entirely a consequence of increased leg length, with little change in trunk length (Tanner et al, 1982). Thus, by implication, greater leg length of testicular cancer cases than their co-twins may be a mark of environmental aetiological factors before puberty, perhaps nutrition, rather than genetic factors, as the reason for an association of height with cancer risk. Height reflects intra-uterine as well as post-natal growth (Proos, 1993), but testicular cancer has been associated with low rather than high birthweight (Swerdlow, 1997), so an association of the malignancy with height seems likely to be of post-natal origin.

An effect of early nutrition on risk of testicular cancer would have some support from published studies. Internationally, testicular cancer incidence correlates well with mean per capita fat intake (Armstrong and Doll, 1975), and fairly well with animal protein, milk and sugar intake, although these analyses were of concurrent national food consumption rather than consumption when the subjects were young. In three Scandinavian countries, lower rates of testicular cancer than would be expected from long-term trends have been observed in cohorts of men who were young during World War II (Møller, 1993; Bergström et al, 1996), which could reflect altered childhood nutrition during the War. Similar effects were not seen, however, in several other Continental European countries (Bergström et al, 1996). In a

*© 1999 Cancer Research Campaign*

case-control study, an association of testicular cancer risk has been found with retrospectively reported milk consumption in adolescence, based on a moderate response rate (Davies et al, 1996). We found no association of risk with childhood dairy product consumption (we did not ask separately about milk), but our results on food intake were weak because few pairs reported any difference.

The several clusters of genitourinary malformations in families of men with testicular cancer, although the data we collected on this is insufficient for formal analysis, add to previous evidence suggesting that familial associations of testicular cancer and such malformations may sometimes have a genetic basis (Tollerud et al, 1985; Forman et al, 1992). In the previous literature, undescended testis does not appear to be substantially more frequent, however, in familial testicular cancer cases than others (Tollerud et al, 1985; Forman et al, 1992; Heimdal et al, 1996).

The testicular cancers in brothers in our study, with the exception of those in the co-twins previously published (three concordant pairs) (Swerdlow et al, 1997), were ascertained from questionnaires, not medical records. The reported diagnoses are likely to be correct, however, for such distinctive malignancies at a young age. There is potential for the presence of malignancy in a brother to have influenced whether the twin responded to our questionnaire, but the effect would be small compared with the risk found. The greatly raised risk of testicular cancer in non-twin brothers of men with the disease accords with previous studies, in which the relative risk has been around 10, while the risk for fathers, in whom no cases were reported in our study, was considerably less raised (Forman et al, 1992; Heimdal et al, 1996; Westergaard et al, 1996). These data on non-twin brothers, with those on risks in twin brothers (Swerdlow et al, 1997) (relative risk of testicular cancer = 37.5 (95% CI 12.3–115.6) for all same-sex co-twins), indicate that the genetic element in risk of testicular cancer is far greater than that for most other cancers (Easton and Peto, 1990).

As in a small previous study of 13 twins with testicular cancer (Braun et al, 1995) there was no evidence that the increased risk of testicular cancer in DZ twins (Braun et al, 1995; Swerdlow et al, 1997) could be the consequence of an increased prevalence of cryptorchidism in the twins: the two cryptorchid men among 40 DZ cases (three out of 41 if the concordant pair are counted as two cases) in our data are less than the 10% of subjects who are cryptorchid among testicular cancer cases generally (Swerdlow, 1997); indeed it was among MZ cases that cryptorchidism was common (five of 20, or six of 22 if concordant pair members were both included), for no obvious reason. Although based on small numbers, the apparent association in our study of cryptorchidism in the twin pairs with lower birthweight parallels such an association in the general population (John Radcliffe Hospital Cryptorchidism Study Group, 1986). The large raised risk of testicular cancer in relation to cryptorchidism in the twins was similar to that seen in case-control and cohort studies of unrelated subjects (Swerdlow, 1997). This is compatible with the theory that cryptorchidism and testicular cancer are associated because the former is a cause of the latter, but does not fit easily with the alternative hypothesis (Henderson et al, 1979) that this association is due to common aetiology of both by in-utero exposure to high concentrations of maternal oestrogens. If this latter hypothesis were correct, one might expect that cryptorchidism would be a diminished or absent risk factor for testicular cancer in twin pairs, since the maternal hormone concentrations would be the same for both the case and control. This argument is not decisive, however: the presence of cryptorchidism was self-reported; placentation can differ between twins, so that maternal hormone exposure might not be identical between them, and the result on cryptorchidism was based on small numbers. For the findings on cryptorchidism and those on height and limb length, further data on twins would be valuable.

### ACKNOWLEDGEMENTS

We are grateful to the Cancer Research Campaign and Medical Research Council for funding. We thank Professor Tom Mack for his advice and encouragement, the Office for National Statistics for cancer and birth data; Helen Lipsey, Augustus Casely-Hayford, Andrew Reid, Ha Nguyen, P Riach, S Armitage, Y Forshaw, and the staff at the NHSCRs for England and Wales, and Scotland for help in identifying the twins and obtaining information about them, and the twins themselves for their time and cooperation.

### REFERENCES

Albanes D, Jones DY, Schatzkin A, Micozzi MS and Taylor PR (1988) Adult stature and risk of cancer. *Cancer Res* **48**: 1658–1662

Armstrong B and Doll R (1975) Environmental factors and cancer incidence and mortality in different countries, with special reference to dietary practices. *Int J Cancer* **15**: 617–631

Bergström R, Adami H-O, Möhner M, Zatonski W, Storm H, Ekbom A, Tretli S, Teppo L, Akre O and Hakulinen T (1996) Increase in testicular cancer incidence in six European countries: a birth cohort phenomenon. *J Natl Cancer Inst* **88**: 727–733

Braun MM, Caporaso NE, Page WF and Hoover RN (1995) Prevalence of a history of testicular cancer in a cohort of elderly twins. *Acta Genet Med Gemellol* **44**: 189–192

Breslow NE and Day NE (1980) *Statistical Methods in Cancer Research, Vol 1, The Analysis of Case-control Studies*. IARC Scientific Publication no 32. IARC: Lyon.

Brown LM, Pottern LM and Hoover RN (1987) Testicular cancer in young men: the search for causes of the epidemic increase in the United States. *J Epidemiol Community Health* **41**: 349–354

Davies TW, Palmer CR, Ruja E and Lipscombe JM (1996) Adolescent milk, dairy product and fruit consumption and testicular cancer. *Br J Cancer* **74**: 657–660

Davies TW, Prener A and Engholm G (1990) Body size and cancer of the testis. *Acta Oncol* **29**: 287–290

Easton D and Peto J (1990) The contribution of inherited predisposition to cancer incidence. *Cancer Surv* **9**: 395–415

Floud R, Wachter K and Gregory A (1990) *Height, Health and History. Nutritional Status in the United Kingdom, 1750–1980*. Cambridge University Press: Cambridge

Forman D, Oliver RTD, Brett AR, March SGE, Moses JH, Bodmer JG, Chilvers CED and Pike MC (1992) Familial testicular cancer: a report of the UK family register, estimation of risk and an HLA class 1 sib-pair analysis. *Br J Cancer* **65**: 255–262

Gallagher RP, Huchcroft S, Phillips N, Hill GB, Coldman AJ, Coppin C and Lee T (1995) Physical activity, medical history, and risk of testicular cancer (Alberta and British Columbia, Canada). *Cancer Causes Control* **6**: 398–406

Heimdal K, Olsson H, Tretli S, Flodgren P, Børresen A-L and Fosså SD (1996) Familial testicular cancer in Norway and southern Sweden. *Br J Cancer* **73**: 964–969

Henderson BE, Benton B, Jing J, Yu MC and Pike MC (1979) Risk factors for cancer of the testis in young men. *Int J Cancer* **23**: 598–602

John Radcliffe Hospital Cryptorchidism Study Group (1986) Cryptorchidism: an apparent substantial increase since 1960. *Br Med J* **293**: 1401–1404

Mack TM, Cozen W, Shibata DK, Weiss LM, Nathwani BN, Hernandez AM, Taylor CR, Hamilton AS, Deapen DM and Rappaport EB (1995) Concordance for Hodgkin's disease in identical twins suggesting genetic susceptibility to the young-adult form of the disease. *N Engl J Med* **332**: 413–418

Møller H (1993) Clues to the aetiology of testicular germ cell tumours from descriptive epidemiology. *Eur Urol* **23**: 8–13

© 1999 Cancer Research Campaign 

Office of Population Censuses and Surveys (1985) *Cancer Statistics Registrations 1982*. Series MBI No 14. HMSO: London

Proos LA (1993) Anthropometry in adolescence – secular trends, adoption, ethnic and environmental differences. *Horm Res* **39**: 18–24

Swerdlow AJ (1997) Epidemiology of testicular cancer. In *Principles and Practice of Genitourinary Oncology*, Raghavan D, Scher HI, Leibel SA, Lange PH (eds), pp. 643–652. Lippincott-Raven: Philadelphia

Swerdlow AJ, Huttly SRA and Smith PG (1989) Testis cancer: post-natal hormonal factors, sexual behaviour and fertility. *Int J Cancer* **43**: 549–553

Swerdlow AJ, De Stavola B, Maconochie N and Siskind V (1996) A population-based study of cancer risk in twins: relationships to birth order and sexes of the twin pair. *Int J Cancer* **67**: 472–478

Swerdlow AJ, De Stavola BL, Swanwick MA and Maconochie NES (1997) Risks of breast and testicular cancers in young adult twins in England and Wales: evidence on prenatal and genetic aetiology. *Lancet* **350**: 1723–1728

Tanner JM (1989) *Foetus into Man. Physical Growth from Conception to Maturity*, 2nd edn. Castlemead: Ware, UK

Tanner JM, Hayashi T, Preece MA and Cameron N (1982) Increase in length of leg relative to trunk in Japanese children and adults from 1957 to 1977: comparison with British and with Japanese Americans. *Ann Hum Biol* **9**: 411–423

Tollerud DJ, Blattner WA, Fraser MC, Brown LM, Pottern L, Shapiro E, Kirkemo A, Shawker TH, Javadpour N, O'Connell K, Stutzman RE and Fraumeni JF Jr (1985) Familial testicular cancer and urogenital developmental anomalies. *Cancer* **55**: 1849–1854

Torgersen S (1979) The determination of twin zygosity by means of a mailed questionnaire. *Acta Genet Med Gemellol* **28**: 225–236

UK Testicular Cancer Study Group (1994) Social, behavioural and medical factors in the aetiology of testicular cancer: results from the UK study. *Br J Cancer* **70**: 513–520

Westergaard T, Olsen JH, Frisch M, Kroman N, Nielsen JW and Melbye M (1996) Cancer risk in fathers and brothers of testicular cancer patients in Denmark. A population-based study. *Int J Cancer* **66**: 627–631

Whittemore AS, Paffenbarger RS Jr, Anderson K and Lee JE (1984) Early precursors of urogenital cancers in former college men. *J Urol* **132**: 1256–1261

*© 1999 Cancer Research Campaign*
